# Quantitative analysis of heat release during coal oxygen-lean combustion in a O_2_/CO_2_/N_2_ atmosphere by TG-DTG-DSC

**DOI:** 10.1038/s41598-022-10752-5

**Published:** 2022-04-23

**Authors:** Jingdong Shi, Hetao Su, Yunzhuo Li, Zijun Huang, Yiru Wang, Lintao Gao

**Affiliations:** 1 School of Engineering and Technology, China University of Geosciences (Beijing), Beijing, 100083 China; 2 Key Laboratory of Deep GeoDrilling Technology, Ministry of Natural Resources, China University of Geosciences (Beijing), Beijing, 100083 China

**Keywords:** Energy science and technology, Fossil fuels

## Abstract

Heat release of coal combustion in an oxygen-lean and multi-gas environment is a common phenomenon, coalfield fires caused by it can lead to serious environmental destruction and loss of coal resources. Simultaneous thermal analysis experiments for Bulianta (BLT, high-volatile bituminous coal) and Yuwu coal (YW, anthracite) in 21vol.%O_2_/79vol.%N_2_ and 15vol.%O_2_/5vol.%CO_2_/80vol.%N_2_ were carried out to study the law of heat release. Based on the TG-DTG-DSC curves, the combustion characteristic parameters were analyzed. Decreasing O_2_ concentration caused a significant reduction of local reactivity and further the decreasing maximum heat release rate for low-rank coal, while increasing CO_2_ concentration caused a significant thermal lag effect and further the increasing maximum heat release rate for high-rank coal. The relationship between the heat release rate and the reaction rate constant was quantitatively analyzed. At the increasing stage of the heat release rate, the heat release rate of the two coals increased conforming to ExpGro1 exponential model. At the decreasing stage of the heat release rate, the heat release rate of YW coal decreased exponentially with the reaction rate constant, while the heat release rate of BLT coal decreased linearly. Regardless of the atmospheres, the conversion rates corresponding to maximum heat release rate of BLT and YW coal were about 0.80 and 0.50, respectively, indicating that the coal rank played a dominant role. The results are helpful to understand the heat release process of coal oxygen-lean combustion in O_2_/CO_2_/N_2_.

## Introduction

Coal is an important energy source to meet the power demand as well as to promote the economy development because of its abundant reserves^1–3^. Coalfield fires triggered by spontaneous coal combustion also occur continuously when mining, and are considered a global crisis, which not only causes serious environmental destruction and loss of coal resources, but also poses a serious threat to human safety and health^4–7^. Most coalfield fires occur in an oxygen-lean (oxygen concentration lower than air) and multi-gas environment due to insufficient oxygen supply and combustion product gases^8^. The development and expansion of coalfield fires closely relate to the heat accumulation of coal combustion. Obtaining the law of heat release during coal oxygen-lean combustion in a multi-gas atmosphere will be beneficial to understand and reveal the dynamic spread of a coalfield fire.

At present, most scholars have carried out a lot of research on the heat release of coal combustion under conventional air combustion. Pan et al.^2^ studied the heat release of the oxidation characteristics of pulverized coal under conventional air combustion using a C600 microcalorimeter. The results showed that the oxidative heat evolution of pulverized coal has obvious stage characteristics of first absorbing heat and then releasing heat. Other scholars^9–12^ also came to a conclusion consistent with the above. Deng et al.^13^ investigated the gas production and thermal behavior of weathered coal and fresh coal. They found that, a significant difference existed in the thermal energy release between weathered coal and fresh coal at different oxidation stages. Su et al.^14^ studied the main characteristic behaviors (temperature gradient, oxygen consumption, oxidation kinetics, gaseous products and heat release) of coal combustion, and divided the evolution process into five stages. Further, heat release during the last three stages was classified into three heat levels. Zhao et al.^15^ divided the high-temperature oxidation process into four stages using thermogravimetric differential scanning calorimetry (TG-DSC), including water evaporation and gas desorption, oxygen absorption and weight gain, thermal decomposition and combustion, and obtained detailed heat release characteristics. Wang et al.^16^ studied the thermal behaviors and kinetic characteristics of coal oxygen-lean combustion at high temperature by TG-DSC synchronous thermal analysis. The results showed that the centralized weight loss and exothermic processes became dispersed in an oxygen-lean atmosphere, and the effects of oxygen concentration enhanced when it was lower than 13 vol.%. There was a linear relationship between mass and heat release, and the relationship between mass and heat release changed in stages with oxygen concentration.

Some studies analyzed the oxygen-lean combustion behaviors of coal in a O_2_/N_2_/CO_2_ atmosphere. Ren et al.^12^ performed coal oxidation and combustion heat behavior analysis experiment in O_2_/N_2_/CO_2_ and O_2_/N_2_ atmospheres (O_2_ concentration of 21%, 14%, 8%, CO_2_ concentration of 0%, 39%, 46%, 52%). The results illustrated that the increase of CO_2_ concentration or the decrease of O_2_ concentration had a delay effect on the TG and DSC curve. Su et al.^17^ studied the dynamical oxygen-lean combustion behaviors of two coal samples in 21vol.%O_2_/79vol.%N_2_ and 15vol.%O_2_/5vol.%CO_2_/80vol.%N_2_ atmospheres by conducting simultaneous thermal analysis. The results showed that there was an ignition delay phenomenon for coal lean-oxygen combustion in the O_2_/CO_2_/N_2_ atmosphere, and apparent activation energy increased at III stage and decreased at IV stage in the O_2_/CO_2_/N_2_ atmosphere, compared with in the O_2_/N_2_ atmosphere. However, they did not further study the heat release of coal oxygen-lean combustion in the O_2_/CO_2_/N_2_ atmosphere. In addition, some studies studied the combustion behavior of coal in the N_2_/CO_2_/O_2_, O_2_/H_2_O/CO_2_ and O_2_/N_2_/H_2_O atmospheres^18–20^. Simultaneously, they did not analyze the heat release of coal oxygen-lean combustion, and the effect of multi-gas atmosphere on the heat release of coal oxygen-lean combustion.

Heat release is the basis of coalfield fire spreading. However, there are currently few studies on the heat release of coal oxygen-lean combustion in a multi-gas environment. The purpose of this work is to analyze the law of heat release during coal oxygen-lean combustion in a O_2_/CO_2_/N_2_ atmosphere. Simultaneous thermal analysis experiments were carried out for two coal samples in 21vol.%O_2_/79vol.%N_2_ and 15vol.%O_2_/5vol.%CO_2_/80vol.%N_2_, respectively. Based on the TG-DTG-DSC curves, the combustion characteristic parameters were discussed, and the kinetic parameters were obtained. Furthermore, the relationship between the exothermic rate and the reaction rate constant was proposed. This work can provide theoretical support for revealing the spread of coalfield fires.

## Experiments and methods

### Preparation of coal samples

Two fresh coal samples were selected from the Bulianta colliery in Inner Mongolia and the Yuwu colliery in Shanxi, China, denoted as BLT and YW, respectively. The reason for choosing these two kinds of coal is that they belong to different rank coals and can show good experimental results. BLT coal belongs to high-volatile bituminous coal, which has a higher volatile matter (31.66%), lower fixed carbon (43.30%) and higher ash content (16.16%) than that of YW coal. YW coal belongs to anthracite. Coal samples were crushed in the laboratory, then sieved through 0.60 mm, 0.45 mm and 0.30 mm gauze. The particle size between 0.30 and 0.45 mm were selected as the experimental coal samples. The proximate analysis and ultimate analysis had been carried out in our previous research^17^, as shown in Table 1**.**

**Table 1:** Proximate analysis and ultimate analysis of coal samples^17^.

Coal sample	Proximate analysis (ad, %)					Ultimate analysis (ad, %)			
	Moisture	Ash	Volatile matter	Fixed Carbon	Nitrogen	Carbon	Hydrogen	Sulfur	Oxygen
BL coal	8.88	16.16	31.66	43.30	0.94	63.44	5.08	0.28	15.03
YW coal	0.71	9.40	9.90	79.99	1.30	83.48	4.05	0.24	3.72

### TG-DTG‑DSC experiment

**Figure 1:**
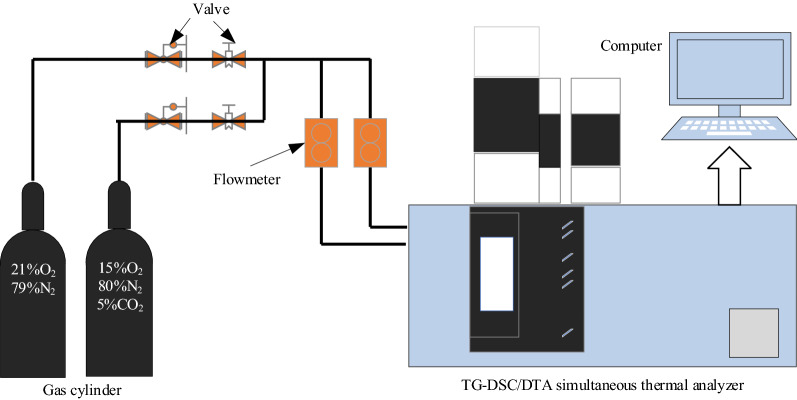
Schematic diagram of the experimental system.

A synchronous thermal analyzer (NETZSCH STA 449 F3) was utilized. According to the detection of gas environment and gas concentration in most coal fire areas in China by scholars, coalfield fires are mostly in an oxygen-lean and multi-gas environment, wherein include an oxygen-lean environment with 15vol.%O_2_^21^. In order to study the dynamical oxygen-lean combustion behaviors of coal in a multi-gas environment, we have chosen 21vol.%O_2_/79vol.%N_2_ and 15vol.%O_2_/5vol.%CO_2_/80vol.%N_2_ atmospheres to conduct simultaneous thermal analysis experiments of two coal samples in our previous research^17^. We continued to choose the above two atmospheres to carry out research in this work, the purpose is to study the law of heat release during coal oxygen-lean combustion in a multi-gas environment based on traditional air combustion. Two atmosphere gases were placed in two cylinders respectively. The coal sample was put in a container. Gases passed into the container from two inlets, one of which located the bottom with a gas flow rate of 50 ml/min and another one located the middle with a gas flow rate of 20 ml/min. Two kinds of coal samples, with a mass of about 13 mg were heated from room temperature to 1100 °C, at three heating rates of 10 °C/min, 15 °C/min, and 20 °C/min, respectively, as seen in Table 2. Based on the synchronous thermal analyzer, the schematic diagram of the experimental system is shown in Fig. 1.

**Table 2:** Experimental design.

Experiment number	Atmosphere	Heating rate (°C/min)	Heating range (°C)
BLT 1	O_2_/N_2_	10	Room temperature ~ 1100
BLT 2	O_2_/N_2_	15	
BLT 3	O_2_/N_2_	20	
BLT 4	O_2_/CO_2_/N_2_	10	
BLT 5	O_2_/CO_2_/N_2_	15	
BLT 6	O_2_/CO_2_/N_2_	20	
YW 1	O_2_/N_2_	10	
YW 2	O_2_/N_2_	15	
YW 3	O_2_/N_2_	20	
YW 4	O_2_/CO_2_/N_2_	10	
YW 5	O_2_/CO_2_/N_2_	15	
YW 6	O_2_/CO_2_/N_2_	20

### Combustion kinetic theory

The coal combustion kinetic equation can be expressed as follows^22^

1 $$\frac{d\alpha }{{dt}} = k\left( T \right) \cdot f\left( \alpha \right)$$

where, *k*(*T*) is the reaction rate constant. *α* corresponds to the conversion of coal, its expression is as follows

2 $$\alpha = \frac{{W_{{\text{O}}} - W{\text{i}}}}{{W_{{\text{O}}} - W_{\infty } }}$$

where, *W*_i_ means the coal mass corresponding to the time of *i*.

The reaction rate constant of coal combustion can be expressed as follows^23^.

3 $$k\left( T \right) = A\exp \left( { - \frac{E}{{{\text{R}}T}}} \right)$$

where, *A* corresponds to the pre-exponential factor (min^−1^); *E* corresponds to the apparent activation energy (kJ/mol), R corresponds to the universal gas constant.

The kinetics equation of non-isothermal reaction can be expressed as follows^24^

4 $$\frac{d\alpha }{{dT}} = \frac{A}{\beta }\exp \left( { - \frac{E}{{{\text{R}}T}}} \right)f\left( \alpha \right)$$

where, *β* corresponds to the heating rate for non-isothermal experiments.

Due to the high accuracy, the Kissinger–Akahira–Sunose (KAS) method was utilized to calculate the apparent activation energy. Its expression is as follows

5 $$\ln \left( {\frac{\beta }{{T_{\alpha }^{2} }}} \right) = \ln \left[ {\frac{AR}{{Eg\left( \alpha \right)}}} \right] - \frac{E}{{{\text{R}}T}}$$

Based on the plot of ln(*β*/*T*_*α*_^2^) versus 1000/T, activation energies were calculated from the slope of the linear regression lines, pre-exponential factors were estimated from the intercepts.

## Results and discussions

### The influence of the O_2_/CO_2_/N_2_ atmosphere on the combustion characteristic parameters

**Figure 2:**
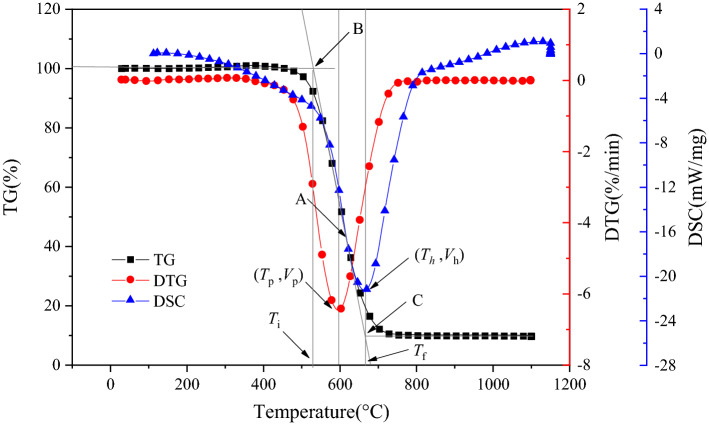
Calculation of combustion characteristic parameters.

Figure 2 gives the calculation method of combustion characteristic parameters, including ignition temperature (*T*_i_), maximum combustion rate (*v*_p_) and the temperature corresponding to maximum combustion rate (*T*_p_), maximum heat release rate (*v*_h_) and the temperature corresponding to maximum heat release rate (*T*_h_), and burnout temperature (*T*_f_). The vertical line passing through the DTG curve point (*T*_p_, *v*_p_) intersects the TG curve at point A, and the tangent line passing through point A intersects the straight line when the TG curve begins to descend at point B. The abscissa of point B corresponds the *T*_i_. Similarly, the abscissa of point C corresponds the *T*_f_. (*T*_p,_ *v*_p_) is valley point on the DTG curve. (*T*_h,_ *v*_h_) is the valley point on the DSC curve. The results obtained are shown in Table 3. When the heating rate was constant, the values of *T*_i_, *T*_p_, *T*_h_ and *T*_f_ in the O_2_/CO_2_/N_2_ atmosphere visibly increased compared with that in the O_2_/N_2_ atmosphere. This indicated that a delay of ignition, heat release and burnout existed during coal oxygen-lean combustion in the O_2_/CO_2_/N_2_ atmosphere. This result was consistent with the literature^17,18,25,26^.

**Table 3:** Characteristic parameters of BLT and YW coal.

Experiment number	*T*_i_ (°C)	*T*_p_ (°C)	*V*_p_(%/min)	*T*_h_ (°C)	*V*_h_(mW/mg)	*T*_f_ (°C)
BLT 1	401.16	472.07	7.65	491.94	32.75	523.38
BLT 2	394.45	474.47	9.85	522.18	41.01	558.60
BLT 3	370.52	485.21	11.76	539.18	45.36	572.82
BLT 4	431.16	511.42	6.92	521.05	30.20	539.82
BLT 5	425.75	531.00	8.32	549.20	36.83	581.42
BLT 6	385.53	476.61	7.89	573.96	28.85	635.36
YW 1	513.40	570.81	7.61	568.39	24.84	631.01
YW 2	519.18	598.00	7.97	608.91	26.68	689.31
YW 3	529.82	617.44	8.54	641.79	29.74	734.06
YW 4	530.42	599.30	6.16	598.98	21.32	675.60
YW 5	546.19	629.17	7.49	636.54	28.11	740.29
YW 6	544.22	649.35	8.63	654.27	35.99	775.56

In the O_2_/CO_2_/N_2_ atmosphere, for BLT coal sample, the values of *v*_p_ decreased by 0.73%/min, 1.53%/min, and 3.87%/min at 10 °C/min, 15 °C/min, and 20 °C/min, respectively, and the values of *v*_h_ decreased by 2.55 mW/mg, 4.18 mW/mg, and 16.51 mW/mg at 10 °C/min, 15 °C/min, and 20 °C/min, respectively, compared with that in the O_2_/N_2_ atmosphere, because the decreasing O_2_ concentration leaded to a reduction of local reactivity^27^. For YW coal sample, the values of *v*_p_ decreased by 1.45%/min, 0.48%/min, and -0.09%/min at 10 °C/min, 15 °C/min, and 20 °C/min, respectively, and the values of *v*_h_ increased by -3.52 mW/mg, 1.43 mW/mg, and 4.25 mW/mg at 10 °C/min, 15 °C/min, and 20 °C/min, respectively. The reason was that coal absorbed enough oxygen at low heating rate and O_2_ played a leading role on the decreasing *v*_p_ and *v*_h_. Decreasing O_2_ concentration leaded to a reduction of local reactivity and further the decreasing maximum heat release rate^27^. At high heating rate, coal absorbed less oxygen and CO_2_ played a leading role on the increasing *v*_h_. Increasing CO_2_ concentration leaded to a thermal lag effect and further the increasing maximum heat release rate^28^. It can be seen that the influence of the O_2_/CO_2_/N_2_ atmosphere on the maximum heat release rate was restricted by the coal rank. The low-rank coal burned faster due to its low carbon content, and O_2_ had a significant impact on the maximum heat release rate. The high-rank coal contained more carbon and burned slowly, and CO_2_ had a significant impact on the maximum heat release rate.

### The influence of the O_2_/CO_2_/N_2_ atmosphere on the kinetic parameters by KAS method

**Figure 3:**
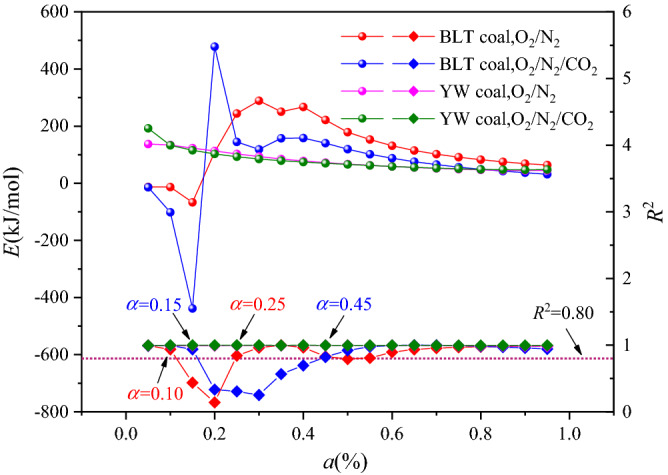
The values of apparent activation energy and *R*^2^ for BLT and YW coal.

Figure 3 shows the changes in the values of apparent activation energy and correlation coefficients (*R*^2^) by KAS method, in the two atmospheres. For BLT coal, as the conversion rate increased, the values of apparent activation energy all first decreased, then increased, and finally decreased. In order to divide the low-temperature oxidation and the combustion stages, the corresponding conversion rate at the *T*_i_ in two atmospheres was calculated respectively. The results showed that, in the O_2_/N_2_ atmosphere, the conversion rate at the *T*_i_ were 0.17, 0.12, 0.14, at 10 °C/min, 15 °C/min, and 20 °C/min, respectively. In the O_2_/CO_2_/N_2_ atmosphere, the conversion rate at the *T*_i_ were 0.15, 0.15, 0.11, at 10 °C/min, 15 °C/min, and 20 °C/min, respectively. Therefore, In the range of 0.5–0.15 conversion rate, three groups of experiments for BLT 1, BLT 4 and BLT 5 were in the low-temperature oxidation process, three groups of experiments for BLT 2, BLT 3 and BLT 6 were in the initial stage of ignition. Since the *R*^2^ was lower than 0.80, the values of apparent activation energy were not accurate and cannot be compared. When the conversion rate was higher than 0.15, the coal sample was ignited, and the values of apparent activation energy values in the two atmospheres appeared a sudden increase. During the combustion process, the values of apparent activation energy in the O_2_/CO_2_/N_2_ atmosphere were approximately 33–58% lower than that in the O_2_/N_2_ atmosphere. This was because the heat released by coal combustion accumulated more easily in the O_2_/CO_2_/N_2_ atmosphere than in the O_2_/N_2_ atmosphere, as a result of the reduction of 6 vol.% O_2_ and the addition of 5 vol.% CO_2_ (low heat conduction coefficient).

For YW coal, as the conversion rate increased, the values of apparent activation energy in the two atmospheres kept decreasing. When the conversion rate was 0.05, it was in the low-temperature oxidation process, and the values of apparent activation energy in the O_2_/CO_2_/N_2_ atmosphere were approximately 40% higher than that the O_2_/N_2_ atmosphere. This has been confirmed in the research of others^17,29^. When the conversion rate was higher than 0.05, the coal was ignited, and the values of apparent activation energy in the two atmospheres were close. The influence of the atmosphere was no longer obvious.

In addition, the *R*^2^ in the two atmospheres was greater than 0.99. However, for BLT coal, a decrease behavior was showed in the conversion rates ranges of 0.10–0.25 in O_2_/N_2_ and 0.15–0.45 in O_2_/CO_2_/N_2_, respectively. The reason was that the precipitation of the remaining volatiles was promoted by the heat release of separated volatiles combustion, and the precipitation and combustion of volatile was significantly deferred in the O_2_/CO_2_/N_2_ atmosphere compared with that in the O_2_/N_2_ atmosphere^27,30,31^, because of the slightly lower diffusivity of volatiles in CO_2_ than in N_2_ and the lower mass flux of oxygen to the volatiles flame^28,32–34^.

### The influence of the O_2_/CO_2_/N_2_ atmosphere on the heat release

The reaction rate between oxygen and coal is the key factor influencing the heat release rate^35^. Studying the relationship between the heat release rate and reaction rate is beneficial to understand in the heat release process during coal oxygen-lean combustion in the O_2_/CO_2_/N_2_ atmosphere, which can provide a theoretical foundation for revealing the law of coalfield fire spreading. Since the value of the reaction rate constant can directly reflect the reaction rate, the reaction rate constant was used instead of the reaction rate in this study. According to our previous research^17^, the kinetic mechanism functions of BLT and YW coal were Jander (Diffusional (3-D)) and three-level chemical reaction, respectively. The values of pre-exponential factor were calculated though Eq. (5), and then the values of reaction rate constant were obtained though Eq. (3).

**Figure 4:**
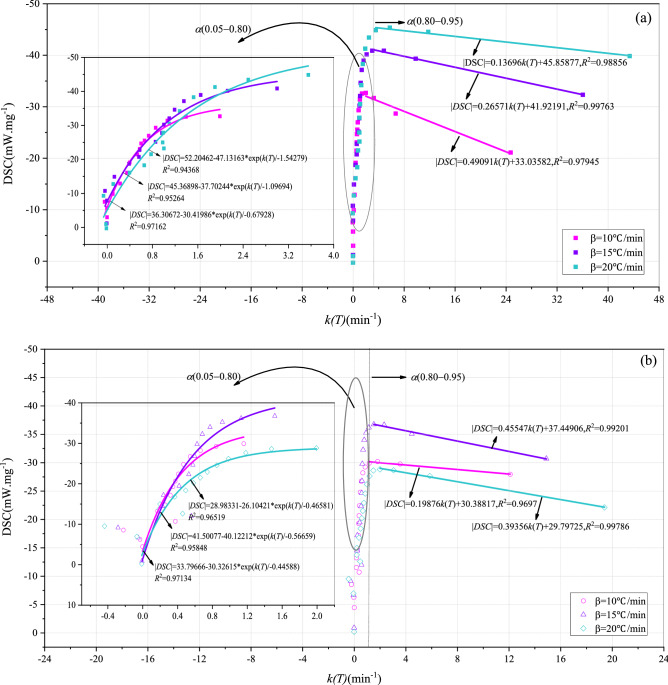
DSC-k(T) curves of BLT coal. (**a**) O_2_/N_2_, (**b**) O_2_/CO_2_/N_2_.

**Figure 5:**
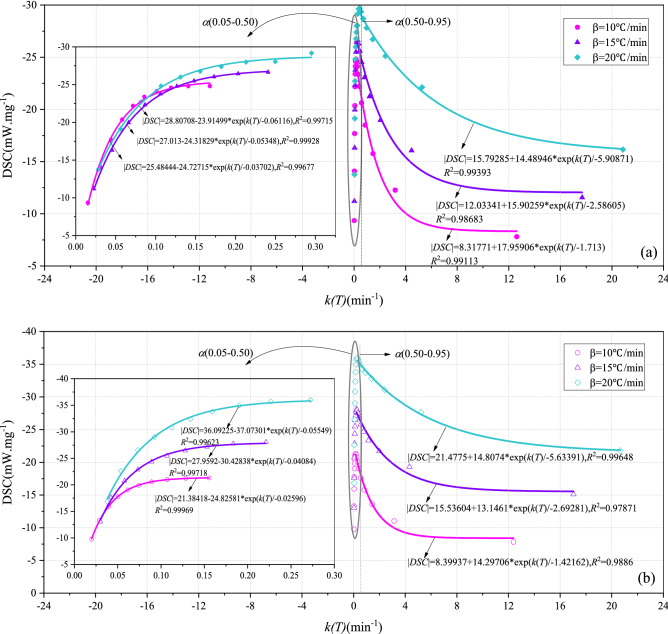
DSC-k(T) curves of YW coal. (**a**) O_2_/N_2_, (**b**) O_2_/CO_2_/N_2_.

Figures 4 and 5 show the DSC-k(T) curves of BLT and YW coal, respectively. The conversion rate corresponding to the maximum heat release rate was taken as a segment point, and the DSC-k(T) curves were divided into two stages: the increasing stage and the decreasing stage of the heat release rate. The conversion rate corresponding to the maximum heat release rate of BLT and YW coal was always about 0.80 and 0.50, respectively., indicating that the conversion rate corresponding to the maximum heat release rate was only related to the coal rank, and not corrected to the atmosphere.

At the increasing stage of the heat release rate, the heat release rate of two coals increased exponentially with the increasing reaction rate constant. At the decreasing stage of the heat release rate, the heat release rate of YW coal decreased exponentially with the increasing reaction rate constant, whereas the heat release rate of BLT coal decreased linearly with the increasing reaction rate constant. This was because BLT has higher volatile content and lower fixed carbon content than YW coal, the more active nature resulted in a slow decrease in the heat release rate. ExpGro1 exponential model (see Eq. (6)) was selected to fit the DSC-k(T) curves at the increasing stage of heat release rate for the two coal samples. The model showed a high degree of fit, with the R^2^ for both BLT and YW coal sample above 0.94. Therefore, the relationship between the heat release rate and reaction rate constant for both BLT and YW coal sample can be effectively expressed by the model. The relationship between the heat release rate and the reaction rate constant is approximately as Eq. (7). This formula reflects the characteristic that the heat release rate varies exponentially with the reaction rate. In the follow-up study, when the reaction rate and the most probable mechanism function of coal are known, this formula can be used to carry out dynamic simulation of heat release during coal oxygen-lean combustion in the O_2_/CO_2_/N_2_ atmosphere.

6 $$y = y_{0} + A_{1} {\text{exp}}\frac{x}{{t_{1} }}$$

7 $$\left| {{\text{DSC}}} \right| = y_{0} + A_{1} {\text{exp}}\frac{d\alpha /dt}{{t_{1} f\left( \alpha \right)}}$$

where, *y*_0_ is the offset. *A*_1_ is the amplitude, *t*_1_ is the width.

**Figure 6:**
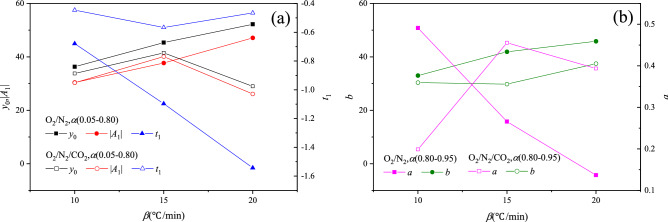
The relationship between *y*_0_, *A*_1_, *t*_1_, *a*, *b* and heating rate of BLT coal. (**a**) *α*(0.05–0.80), (**b**) *α*(0.80–0.95).

**Figure 7:**
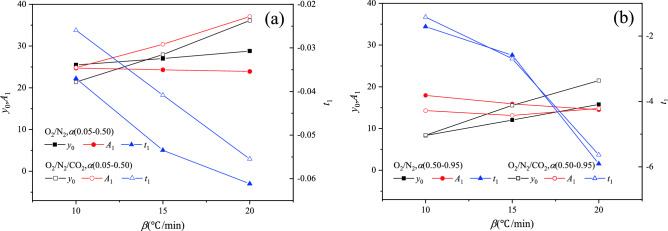
The relationship between *y*_0_, *A*_1_, *t*_1_ and heating rate of YW coal. **a** *α*(0.05–0.50), **b** *α*(0.50–0.95).

In order to quantitatively analyze the relationship between *y*_0_, *A*_1_ and *t*_1_ and heating rate, Figs. 6 and 7 show the changes in *y*_0_, *A*_1_ and *t*_1_ with the heating rate, respectively. There was a linear relationship between *y*_0_, *A*_1_, *t*_1_ and heating rate for YW coal. For BLT coal, *y*_0_, *A*_1_ and *t*_1_ were basically linear with the heating rate in the O_2_/N_2_ atmosphere, whereas there was a non-linear relationship between *y*_0_, *A*_1_, *t*_1_ and heating rate in the O_2_/CO_2_/N_2_ atmosphere. Furthermore, y_0_−β, y_0_−A_1_ and y_0_−t_1_ curves were fitted respectively. The reaction rate constant was calculated using Eq. (1). On the whole, there was a following relationship between the heat release rate and reaction rate of coal, as follows

8 $$\left| {{\text{DSC}}} \right| = a_{1} \beta + b_{1} + \left( {a_{2} \beta + b_{2} } \right) \times {\text{exp}}\frac{d\alpha /dt}{{\left( {a_{3} \beta + b_{3} } \right)f\left( \alpha \right)}}$$

where, *a*_1_ and *b*_1_ are constants related to *y*_0_*, a*_2_ and *b*_2_ are constants related to *A*_1_, *a*_3_ and *b*_3_ are constants related to *t*_1_, as seen in Table 4.

**Table 4:** The value of constants in Eq. (8).

Coal samples	Atmosphere	Conversion rate range	*a*_1_	*b*_1_	*a*_2_	*b*_2_	*a*_3_	*b*_3_
BLT	O_2_/N_2_	0.05–0.80	1.59	20.78	−1.67	−13.35	−0.09	0.19
		0.80–0.95	1.28	21.04	−0.04	0.83	/	/
YW	O_2_/N_2_	0.05–0.50	0.33	22.12	0.08	−25.54	−0.00	−0.01
		0.50–0.95	0.75	0.84	−0.35	21.32	−0.42	2.89
	O_2_/CO_2_/N_2_	0.05–0.50	1.47	6.42	−1.23	−12.41	−0.00	0.00
		0.50–0.95	1.31	−4.48	0.05	13.32	−0.42	3.07

## Conclusions

In this work, simultaneous thermal analysis experiments for BLT coal (high-volatile bituminous coal) and YW coal (anthracite) in the 21%O_2_/79%N_2_ and 15%O_2_/5%CO_2_/80%N_2_ atmospheres were carried out. Based on the TG-DTG-DSC curves, the combustion characteristic parameters were discussed, the values of apparent activation energy were obtained using KAS method, and the relationship between the heat release rate and reaction rate constant was quantitatively analyzed. The following conclusions can be drawn:

1. A delay of ignition and heat release existed during the coal oxygen-lean combustion in O_2_/CO_2_/N_2_. Decreasing O_2_ concentration caused a significant reduction of local reactivity and further the decreasing maximum heat release rate for low-rank coal, while increasing CO_2_ concentration caused a significant thermal lag effect and further the increasing maximum heat release rate for high-rank coal.
2. During the combustion process, the values of apparent activation energy in the O_2_/CO_2_/N_2_ atmosphere were approximately 33–58% lower than that in the O_2_/N_2_ atmosphere for BLT coal, while the values of apparent activation energy in the two atmospheres for YW coal were close. For BLT coal, the values of correlation coefficients were less than 0.80 in the conversion rates ranges of 0.10–0.25 in O_2_/N_2_ and 0.15–0.45 O_2_/CO_2_/N_2_, respectively, which was because that the precipitation of the remaining volatiles was promoted by the heat release of separated volatiles combustion, and the precipitation and combustion of volatile was significantly deferred in the O_2_/CO_2_/N_2_ atmosphere compared with that in the O_2_/N_2_ atmosphere due to the slightly lower diffusivity of volatiles in CO_2_ than in N_2_ and the lower mass flux of oxygen to the volatiles flame.
3. Regardless of the atmospheres, the conversion rates corresponding to maximum heat release rate of BLT and YW coal were about 0.80 and 0.50, respectively, indicating that the coal rank played a dominant role. At the increasing stage of the heat release rate, the heat release rate of the two coals increased conforming to ExpGro1 exponential model. At the decreasing stage of the heat release rate, the heat release rate of YW coal decreased exponentially with the reaction rate constant, while the heat release rate of BLT coal decreased linearly.
